# Langya virus outbreak: current challenges and lesson learned from previous henipavirus outbreaks in China, Australia, and Southeast Asia

**DOI:** 10.1186/s42269-023-01064-3

**Published:** 2023-06-13

**Authors:** Ridwan Olamilekan Adesola, Adriana Viola Miranda, Yeuk Shun Joshua Tran, Ibrahim Idris, Xu Lin, M. B. N. Kouwenhoven, Don Eliseo Lucero-Prisno

**Affiliations:** 1 Department of Veterinary Medicine, Faculty of Veterinary Medicine, University of Ibadan, Ibadan, Nigeria; 2 Global Heath Focus Asia, Bandung, Indonesia; 3 Faculty of Medicine, The Chinese University of Hong Kong, Sha Tin, Hong Kong; 4 Department of Veterinary Medicine, Faculty of Veterinary Medicine, Usmanu Danfodiyo University, Sokoto, Nigeria; 5 Department of Thoracic Surgery, The First Affiliated Hospital, School of Medicine, Zhejiang University, Hangzhou, Zhejiang China; 6 Department of Physics, Xi’an Jiaotong-Liverpool University, Suzhou, China; 7 Department of Global Health and Development, London School of Hygiene and Tropical Medicine, London, UK

**Keywords:** Langya virus, China, *Paramyxoviridae*

## Abstract

**Background and aims:**

A new novel virus, Langya virus (LayV), was detected in China in August 2022, 3 years after the COVID-19 pandemic. LayV is similar to the previously discovered Mojiang henipavirus. Other zoonotic henipaviruses include the Hendra and Nipah viruses. The emergence of the zoonotic Langya virus is attributed to climate change and wildlife encroachment, as LayV is detected in shrews. Those who are infected in China showed various symptoms, but no deaths have been recorded yet. This review aims to shed light on the current state of Langya virus outbreak, its infection control efforts, and the remaining challenges that need to be addressed to curb the outbreak.

**Methods:**

We utilized online publication databases such as PubMed, Google Scholar, and Scopus in writing this review article.

**Results:**

A surveillance study on thirty-five febrile patients in Eastern China identified the Langya virus outbreak. The current efforts from the Chinese government and health authorities to reduce the transmission and spread of Langya virus such as isolation and characterization of LayV, challenges associated with the increase in cases of LayV, and trackable recommendations such as strengthening the healthcare system in China, sensitization of people about risks associated with Langya virus outbreaks, creating an intensive surveillance system network, etc. were discussed.

**Conclusion:**

It is germane and pertinent that the Chinese government and health authorities continue to intensify efforts against Langya virus and address the challenges to effectively reduce transmission.

## Background

The world has been struggling to avert the spread of infectious diseases that seriously threaten global health since 2019, following the outbreak of COVID-19 in Wuhan, China (Tabari et al. 2020). China reported another zoonotic febrile disease on the 4th of August 2022 (Zhang et al. 2022), caused by the Langya virus (LayV), which belongs to the family *Paramyxoviridae*. Studies conducted by a group of scientists show that the LayV genome is closely related to the previously discovered Mojiang henipavirus. Hendra and Nipah viruses are other zoonotic henipaviruses (Chakraborty et al. 2022), that are also related to LayV but the pathogenicity of the viruses differs from one another. Both Hendra virus (HeV) and Nipah virus (NiV) have previously led to deadly outbreaks in Australia and Southeast Asia, respectively (Table 1) (Tabassum et al. 2022). LayV was first identified by researchers in eastern Chinese areas of Shandong and Henan while monitoring febrile patients in three hospitals from April 2018 to August 2021 (Zhang et al. 2022). The genome of LayV was sequenced from a nasopharyngeal sample collected from an infected patient. LayV was then named after a town in Shandong. As of 5 October 2022, no cases of LayV have been detected outside China (Zhang et al. 2022).

**Table 1:** Trends of the outbreak of henipaviruses in China and Southeast Asia

Species of henipavirus	Year	Countries affected	Outbreak to humans	Source of transmission	References
Nipah virus	1998	Malaysia, Singapore	Yes	Pig	Soman Pillai et al. (2020)
	2001	Bangladesh, India	Yes	Date palm sap (Bangladesh), Nosocomial (India)	Soman Pillai et al. (2020)
	2003	Bangladesh	Yes		Soman Pillai et al. (2020)
	2007	India	Yes		Soman Pillai et al. (2020)
	2018	India	Yes	Bats	Soman Pillai et al. (2020)
	2019	India	Yes	Bats	Soman Pillai et al. (2020)
	2000	Cambodia	No	Bats	Reynes et al. (2005)
	2002–2004	Thailand	No	Bats	Wacharapluesadee et al. (2005)
	2008	Indonesia	No	Bats	Sendow et al. (2010)
	2007–2008	Vietnam	No	Bats	Hasebe et al. (2012)
Hendra virus	1994–2022	Australia	No	Horse	Business Queensland (2023)
Langya virus	2022	China	Yes	Shrews	Sah et al. (2022)

Langya virus affects multiple systems. Clinical symptoms and signs of the virus include fever, cough, headache, myalgia, nausea, and vomiting, as well as other blood abnormalities including leukopenia and thrombocytopenia. Apart from that, liver and kidney function are also affected (Lee et al. 2020). At present, no drugs or vaccines are available for LayV infection. Current management of LayV is only through supportive care for the clinical symptoms. This is similar to the treatment for Hendra and Nipah viruses in the past, although treatment trials of ribavirin and chloroquine were since reported to be effective for HeV and NiV infections. These antivirals can also potentially be used for LayV infections since they are related genotypically (Chakraborty et al. 2022).

In this article, we discuss the outbreak of the LayV, current efforts of the Chinese government and health authority, challenges encountered, and recommendations to overcome the challenges.

## Biological characteristics of the Langya virus

The Langya virus is an RNA virus comprising 18,402 nucleotides (Zhang et al. 2022). Considering the nature of LayV as an RNA virus, its mutative potential should be considered with caution. It has a similar genomic structure to other henipaviruses, particularly the Nipah and Hendra viruses, both classified as BSL-4 (biosafety level 4) pathogens (Zhang et al. 2022; Chakraborty et al. 2022) . Phylogenetically, it is most closely related to Mojiang henipavirus, which was found in 2012 in China and was attributed as the cause of three fatal pneumonia cases (Zhang et al. 2022; Wu et al. 2014).

**Fig. 1:**
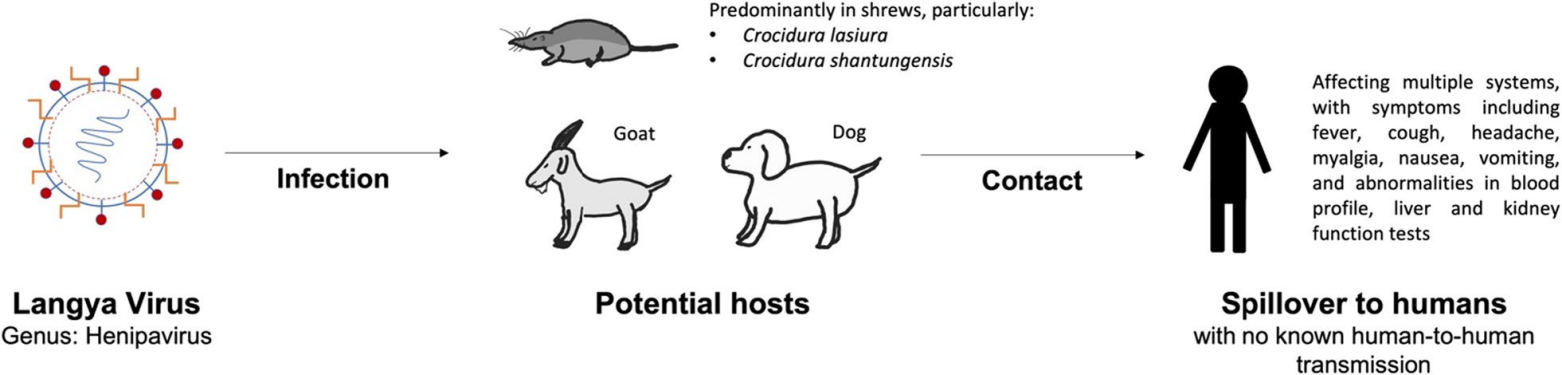
Transmission of Langya virus

The transmission is thought to occur from animals to humans, with no human-to-human transmission reported. A serosurvey of domestic animals found that the viral RNA of LayV was predominantly detected in shrews, specifically the *Crocidura lasiura* shrews commonly found in Northeast Asia. Out of the 121 tested *Crocidura lasiura* shrews, 52.1% had a positive result for LayV. The virus is also found in 20% of the *Crocidura shantungensis* samples, 5% of dogs and 2% of domestic goats surveyed, suggesting multiple potential hosts, with shrews potentially being the natural reservoir of LayV (Fig. 1). The viral load in LayV-infected patients who developed pneumonia was significantly higher in both their swab and serum samples compared to those without pneumonia (Zhang et al. 2022).

## Current efforts

A surveillance study on thirty-five febrile patients in Eastern China identified the Langya virus outbreak. Further investigation reported that among these thirty-five patients, twenty-six were isolated LayV-positive cases (Sah et al. 2022). Despite earlier detection of 14 cases of LayV in 2019, investigations were promptly halted due to COVID-19.

During the LayV outbreak, researchers characterized the virus genome from one of the symptomatic patients, who are mostly farmers and factory workers (Zhang et al. 2022; Chakraborty et al. 2022). This enabled genomic comparison between LayV and other henipaviruses to identify the unknown virus through multiple sequence alignment and phylogenetic tree. A further test was done on the contact-traced people and patients’ family members to confirm the spread of LayV to these people, but they tested negative. However, all family members were negative, suggesting that human-to-human transmission is unlikely (Chakraborty et al. 2022). Subsequently, animals that were living with the patient, such as dogs, goats, and shrews were also tested to determine whether the virus is zoonotic. A higher percentage of LayV antibodies were found in shrews compared to other animals, suggesting that shrews might be the reservoir host of the virus. Despite finding the potential LayV reservoir host, the transmission method remains unknown. Although the Chinese government and health authorities are working together to fight this newly discovered henipavirus, further research is needed by researchers to uncover the mode of transmission and treatment methods for LayV patients.

## Challenges

Currently, very few reports on the Langya virus have reached the public, likely shadowed by the continuous, though subsiding, nationwide attention on COVID-19. Though specific national challenges faced by the Chinese authorities and people remain unknown, other challenges from the limited reports can be identified.

Nationally, the coordination framework of Chinese healthcare may hamper the outbreak response. Looking back at the early stages of the COVID-19 pandemic response, inflexible coordination and inadequate national administrative mandates impeded effective control. Local reports were suppressed and redundantly analyzed across the multi-level health system when they did reach the wider community (Mallapaty 2022).

Locally, health workers and authorities find it difficult to detect the source and prevent the transmission of LayV (Chakraborty et al. 2022). This poses a large issue concerning the prevention of virus transmission. Though no human-to-human transmission has been reported so far, uncertainty lingers when the fundamental of epidemiology remains unknown. This should be a major focus to effectively curb LayV transmission. In addition, there are no approved test kits to detect LayV, meaning patients may have to present with severe symptoms before receiving appropriate treatment. Moreover, routine surveillance of the Langya virus is limited, likely causing a compounding effect with the lack of test kits. Although China is known for its world-class healthcare facility, time is needed before China can develop a robust system to combat the virus.

Apart from public health challenges, scientifically, the biology of the LayV is still poorly understood despite its similarities to the previously discovered henipaviruses. It is imperative for researchers to gain an understanding of LayV’s molecular structure and characteristics. This is crucial in providing essential information for the development of effective therapy and vaccines against the infection as well as to design preventive measures and controls nationally and globally.

## Lesson learned from previous outbreaks of henipavirus in Australia and Southeast Asia

Learning from previous outbreaks will allow for more robust emergency responses. Scrutinizing responses to previous henipaviruses outbreaks allow best practices to be identified, which then can be utilized for LayV control.

The first case of HeV was discovered on August 1st, 1994 in Australia in a 10-year-old mare (Sun et al. 2021). It caused an outbreak of severe acute respiratory diseases among horses that resulted in a high mortality rate. The infection spread to seven additional horses and one human, but they all escaped death. Equine morbillivirus, now known as HeV, is a new paramyxovirus that was discovered to be the cause of the disease. However, the first known instance of HeV was a few months earlier, when there were occurrences of HeV infection in horses; a person who helped with the necropsy of the animals had severe encephalitis and passed away 13 months later. Since HeV was initially discovered in Australia, 62 different HeV outbreaks have occurred there, killing 104 horses either directly or through euthanasia. In addition, HeV has been linked to a total of seven human cases, four of which have resulted in fatalities, with each human infection coming from a horse that had shed the virus (Gazal et al. 2022).

The first outbreak of NiV was detected from 1998 to 1999 in Malaysia and Singapore (Mazzola and Kelly-Cirino 2019). Additionally, NiV outbreaks have been documented in India, with the greatest one occurring in Siliguri, West Bengal in 2001 (about 66 cases and 45 deaths), and a lesser epidemic in Nadia district, West Bengal, in 2007 (five cases with a 100% fatality rate) (Gazal et al. 2022). These outbreaks happened in Bangladesh's Nipah belt, but more recently, in 2018, a NiV outbreak happened in Kerala's Kozhikode and Malappuram districts, which is a part of South India that is geographically apart from the regions impacted by earlier outbreaks. In Kerala's Ernakulum district, one NiV case was identified in 2019. More recently, on September 1, 2021, NiV encephalitis was identified in a 12-year-old child in Kozhikode, Kerala, who passed away on September 5, 2021 (Gazal et al. 2022). The Nipah virus, known to cause encephalitis and acute respiratory distress in humans, is one of the world’s deadliest viruses due to its high mortality rate (39–91%). Over a hundred human deaths were recorded, while over a million infected pigs were euthanized in the 1998 to 1999 outbreak (Field et al. 2010).

A One Health approach was employed during the Hendra and Nipah outbreak to develop preventive measures. The approach is defined as a collaborative and integrated approach to attaining optimal health for people, animals, and the environment. For instance, in Australia, a program to vaccinate horses against the Hendra virus to prevent transmission to humans is currently in place (Manyweathers et al. 2017). Similarly, various stakeholders from the health, agricultural, and animal husbandry sectors were involved in the management of the 2018 and 2019 Nipah virus outbreaks in Kerala through surveillance of livestock, such as farm pigs and bats (Singhai et al. 2021). Additionally, the government of Kerala also strengthen its health emergency response capacity. State-level laboratories were equipped with advanced biocontainment facilities, surveillance was conducted to identify and isolate close contacts, and media outlets and general public were briefed by authorities to prevent fearmongering and help disseminate credible information (Singhai et al. 2021).

These approaches should be considered for the control of the Langya virus. However, the One Health approach faces challenges of its own: for instance, the uptake of the 2012 Hendra vaccine has been slow due to concerns about safety, cost, and effectiveness (Manyweathers et al. 2017).

## Recommendation

**Fig. 2:**
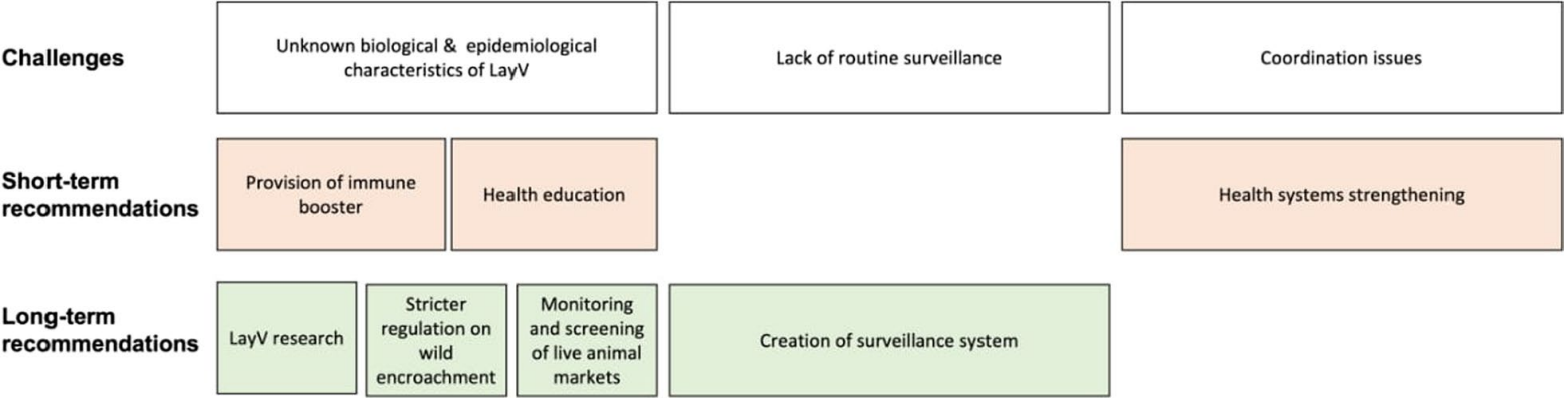
Recommendations for Langya virus control

One Health is a major approach that is being used presently to control zoonotic diseases globally. It is crucial for experts in public health, animal, human, and environmental health as well as other pertinent disciplines and industries to collaborate in order to use a holistic idea to combat LayV in China. We recommend the Chinese government work closely with the China CDC and other stakeholders and employ swift responses based on their recommendations. The reports of redundancy, which was due to miscommunication and underreporting, seen during the COVID-19 pandemic should be avoided by utilizing national emergency notifications. We also recommend the government actively support research, surveillance, monitoring, and health system strengthening. Further recommendations are discussed below to tackle LayV outbreak in a timely manner (Fig. 2).

### Short-term

*Strengthening of healthcare system in China:* The healthcare system is one of the major parastatals that are important in preventing and controlling infections. This includes the provision of adequate personal protective equipment to healthcare workers, the education and training of professionals involved in handling and managing samples, and improving health system capacity.

*Sensitization of people about risks associated with LayV outbreak:* Community health workers should be engaged in educating people on the effects of wildlife encroachment, climate change, disease prevention and control, and one health approach and its importance. This will help in pre-informing the public about risks associated with LayV outbreaks and will prevent further spread of the disease.

*Creation of an intensive surveillance system network in China:* The health authorities should create a stronger community-based LayV surveillance network within the country. This will reduce the transmission of the virus and may also reduce other infectious diseases in China. Other countries in the southeastern part of Asia will also benefit positively from the surveillance network to keep track of henipaviruses outbreaks.

*Provision of immune boosters by the health practitioners:* During this period of LayV outbreak, health practitioners should include immune boosters such as zinc, selenium, vitamin C and D in the supportive care given to the patient with LayV to alleviate the progression of the disease. A study reported that immunocompromised patients infected with LayV showed more severe symptoms compared to non-immunocompromised patients (Shakoor et al. 2021). The immune booster foods should also be encouraged for everyone in order to their immune system against diseases like LayV.

### Long-term

*Engagement in long-term LayV research:* We recommend more research on the mode of transmission of LayV. Researchers can investigate already discovered companion viruses, HeV and NiV, to know more about LayV. This will contribute to the development of specific preventive measures against LayV. After the intensive research work on the characterization of LayV, pathophysiology studies, and vaccine research should not be left behind. Vaccine development should be considered. At present, a clinical trial is being conducted by the National Institute of Allergy and Infectious Disease to evaluate a Nipah vaccine’s ability to prevent infection (Sah et al. 2022). If this trial is successful, a similar platform can also be utilized for LayV. The Chinese government should support these projects through funding and technology support.

*Enforcement of strict regulatory measures on wild encroachment:* As the virus was identified and isolated in shrews, the government should enact strict regulatory measures for wildlife hunting to prevent further LayV outbreaks and the potential outbreak of another novel virus. As the source of the virus remains unknown, the reservoir of the virus should also be closely regulated.

*Monitoring and screening of live animal markets for LayV:* We recommend that animal markets should be carefully monitored in cities and in rural communities where farmers may practice subsistence agriculture. In addition, during this outbreak, the public should avoid places where shrews, the currently identified reservoir, are located, such as markets, zoos, and game reserves. Apart from China, all neighboring countries should monitor the spread of disease on the border to prevent regional transmission of LayV.

## Conclusions

The current efforts from the Chinese government and health authorities to reduce the transmission and spread of LayV such as isolation and characterization of LayV are in place but not enough to eradicate the virus. Trackable recommendations such as strengthening the healthcare system in China, sensitization of people about risks associated with LayV outbreaks, creating an intensive surveillance system network, provision of immune boosters by health practitioners, engaging in long-term LayV research, enforcement of strict regulatory measures on wild encroachment, and monitoring and screening of live animal markets for LayV are provided in this piece to intensify the current efforts provided by the Chinese government and health authorities. It is crucial and germane for the Chinese government and health authorities to continue intensifying efforts with the recommendations provided in this piece and previous lessons learned from the control of other henipaviruses, to effectively reduce the spreading of the Langya virus and avoid further regional outbreaks.

## Data Availability

Not applicable.

## Abbreviations

LayV: Langya virus
COVID-19: Coronavirus disease
HeV: Hendra virus
NiV: Nipah virus
CDC: Centers for disease control and prevention
